# Factors influencing the adoption of HIV prevention measures in low socio-economic communities of inner-city Durban, South Africa

**DOI:** 10.1080/17290376.2023.2185806

**Published:** 2023-03-07

**Authors:** Firoza Haffejee, Jennifer Ducray, Jyotika Basdav, Colette Kell

**Affiliations:** Department of Basic Medical Sciences, Faculty of Health Sciences, Durban University of Technology, Durban, South Africa

**Keywords:** HIV knowledge, risky sexual behaviour, socio-economic factors, South Africa, vulnerable communities

## Abstract

South Africa is the epicentre of the HIV pandemic. Although there have been health promotion education campaigns to reduce HIV incidence, these have not achieved the desired outcomes. When exploring the effectiveness of these campaigns, it is useful not only to examine HIV knowledge, but also to explore the relationship between that knowledge and health-related behaviour. This study aimed to determine the (1) level of knowledge of HIV prevention, (2) relationship between the level of knowledge and the adoption of these behaviours and (3) barriers to sexual behaviour change of vulnerable women in Durban’s city centre, KwaZulu-Natal, South Africa. A mixed methods approach was used to collect information from a marginalised population of women (*n *= 109) attending a non-governmental organisation, which provides for the needs of people from low socio-economic strata. Data were collected during September 2018 at a wellness day programme at the centre. A total of 109 women, over the age of 18 years answered the questionnaire. Knowledge of HIV transmission was high, with majority of participants correctly identifying modes of transmission. Almost all the participants (91.2%) had been tested for HIV, with 68.8% tested a minimum of three times. Despite this, sexual risk behaviour was high. Despite the high level of knowledge of HIV transmission, there was no relationship between HIV knowledge and adoption of behaviours for the prevention of HIV transmission (*p *= .457). However, bivariate analysis showed an association between transactional sex and living in informal housing (OR = 31.94, 95% CI: 5.65–180.63, *p *< .001). Living in informal housing was also associated with having multiple current sexual partners (OR = 6.30, 95% CI: 1.39–28.42, *p *= .02). Multivariate analysis, after adjusting for all other factors, indicated that the odds of having transactional sex was increased by 23 times in those who did not have formal housing (OR = 23.306, 95% CI: 3.97–144.59, *p *= .001). Qualitative responses showed that women perceived poverty as the overarching factor determining the lifestyle choices which impacted their health. They indicated a need for employment opportunities and provision of housing to alleviate both poverty as well as transactional sex. Although, participants from this study understood the benefits of the protective behaviours to prevent HIV transmission, economic and social factors do not afford this vulnerable group the opportunity nor the motivation to adopt such behaviours. In the current climate of increasing unemployment and escalating GBV, urgent interventions are needed in terms of employment opportunities and empowerment drives to prevent an increase in HIV transmission.

## Introduction

South Africa remains the epicentre of the HIV pandemic, accounting for 20% of people living with HIV globally. In the province of KwaZulu-Natal, the prevalence is 16.9% and within some communities in this province 60% of women have HIV (Allinder & Fleischman, 2019). Prevalence rates are particularly high with key population groups which include female sex workers, adolescent girls and young women (AGYW) as well as those aged 15–49 years (AVERT, 2020; Statistics South Africa, 2019; UNAIDS, 2019). Other population groups affected to a lesser extent are men who have sex with men, people who inject drugs and transgender individuals (Cloete, Wabiri, Savva, Van der Merwe, & Simbayi, 2019; UNAIDS, 2019).

In response to the HIV epidemic, the National Department of Health (NDOH) commenced a programme to provide country-wide access to HIV prevention, screening, treatment and support. This programme included HIV education with a view to preventing transmission (Department of Health: Republic of South Africa, 2016). HIV prevention programmes include prevention-of-mother-to-child transmission, condoms, voluntary male circumcision and pre-exposure prophylaxis (PrEP) (AVERT, 2020). More recently, the DREAMS (Determined, Resilient, Empowered, AIDS-free, Mentored and Safe) initiative has been adopted to assist AGYW in combating the HIV pandemic (USAIDS, 2021). Platforms such as LoveLife, Soul City Institute and MTV Shuga provide targeted awareness to young individuals (AVERT, 2020), however, as outlined in the South African Strategic Plan target-specific approaches are required to make further strides in reducing the incidence of HIV (SANAC, 2017).

Within the South African context, health promotion through education is reflected in the proposed South African National Health Insurance (NHI) Bill, as well as the South African Department of Health 2014–2019 strategic plan, the latter stating their mission as improving health status ‘through the prevention of illness and the promotion of healthy lifestyles’ (National Department of Health, 2015). National government has been assisted in these endeavours by LoveLife, a non-profit organisation (NPO) focused on disseminating information through education and awareness campaigns. This includes partnerships with community-based organisations and schools across South Africa, with access to approximately 1.5 million young people (LoveLife, 2020).

Many studies report continued risky behaviour despite suitable HIV health education campaigns (Agarwal, de Araujo, & Paudel, 2013; Cawley et al., 2014; Woodward et al., 2014). Low non-HIV life expectancy and barriers linked to the African cultural context are suggested reasons for this disconnect (Oster, 2012). Cultural factors include the economic dependence on men for survival and subsequent imbalance in power that prevents women from determining their own safe sex practice (Haffejee & Maksudi, 2020; Loggerenberg et al., 2012; Saasa, Choi, & Nackerud, 2018). Stigma also has a negative impact in HIV prevention and testing (Haffejee, Maughan-Brown, Buthelezi, & Kharsany, 2018a) and young people have reported facing stigma when trying to access healthcare facilities (Bernays, Asiimwe, Tumwesige, & Seeley, 2021). An earlier study by Hallman (2005) indicates safe sexual practices are not given importance by those who reside in low-income households. However, discussion of safe sex practices between partners, including those in low socio-economic communities, results in higher rates of condom use (Conserve, Middelkoop, King, & Bekker, 2016).

Over the years, HIV knowledge of prevention and transmission modes has shown great improvement among various geographical districts in South Africa when compared to earlier studies (Mangena-Netshikweta, Maluleke, Maputle, & Mushaphi, 2012; Paul, Ayo, & Ekundayo, 2014; Peltzer, Mngqundaniso, & Petros, 2006). People living in rural areas have low level of knowledge, particularly of HIV transmission (Asefa & Beyene, 2013). While the overall knowledge of HIV is somewhat higher in low socio-economic urban areas, many myths about transmission and prevention exist (Haffejee, Ports, & Mosavel, 2016). Furthermore, adequate knowledge of this, does not always translate into adopting safe sexual practices (Orukwowu, George, & Ene-Peter, 2022). There is a paucity of data on the knowledge of HIV transmission and adoption of preventative strategies amongst vulnerable communities, such as refugees and homeless people within the province of KwaZulu-Natal.

Against this backdrop, this study aimed to determine the (1) level of knowledge of HIV prevention, (2) relationship between the level of knowledge and the adoption of appropriate preventative behaviours and (3) possible barriers to sexual behaviour change of vulnerable women in Durban’s city centre, KwaZulu-Natal, South Africa.

## Methods

This study used a mixed methods approach to collect information from a marginalised population of women attending the Denis Hurley Centre (DHC). Located within the central business district in Durban, South Africa, the DHC is a non-governmental organisation (NGO), which provides for the needs of the homeless, refugees and others of low socio-economic strata. Basic hygiene, meals and healthcare are provided by the centre to these communities, with an estimated population of approximately 150 women who regularly attend the centre for their needs. A wellness day targeting these women was held at the centre on 12–13 September 2018. The programme was advertised to all women who attend the centre. Details of this programme have been elaborated on previously (Ducray, Kell, Basdav, & Haffejee, 2021).

Using the population of 150 women, who attend the centre for their needs, a 95% confidence interval and a 5% margin of error, a minimum sample size of 109 was calculated. Purposive sampling was utilised to recruit women, over the age of 18 years, who attended the wellness day programme.

This report forms part of a larger study, where a questionnaire comprising primarily of 183 questions was utilised.

Quantitative data were collected via 31 close-ended questions, which provided information on HIV knowledge, transmission, testing, prevention and risky behavioural practices. Basic general knowledge of HIV was assessed by asking participants to indicate whether statements regarding HIV were true or false. Furthermore, by allocating a point for each correct answer, a HIV knowledge score was calculated for each participant. Personal behavioural practices, with a focus on risky as well as preventative behaviours, were evaluated by responses to questions on age of sexual debut, sexual partner concurrency, transactional sex and condom use (with consistent use being defined as use at every sexual encounter in the last year). Once again, by allocating points for safe sexual practices and deducting points for risky sexual practices, a HIV prevention score was calculated for every participant. Questions related to gender-based violence such as any history of abuse and transactional sex were also included in order to help elucidate potential barriers to safe sexual practices. Responses were expressed as percentages of the population.

The qualitative data were collected via two open-ended questions: (1) What more do you think could be done to improve the health of women in Durban? (2) Is there anything else related to the topic that we discussed today, that you would like to add to? The responses to these open-ended questions contributed to an in-depth understanding of the lived experiences and challenges underpinning the risky sexual choices reported by some participants. Details of the formerly validated questionnaire were previously published (Haffejee et al., 2016; Ports, Haffejee, Mosavel, & Rameshbabu, 2015). The questionnaire was available in both English and isiZulu, the predominant languages of the region.

Due to participant literacy constraints, the questionnaire was administered by research assistants fluent in both languages. A total of seven research assistants were included; five were female and two were male. Bias was minimised by training research assistants and questions were asked exactly as depicted on the questionnaire and responses were scribed verbatim. The questionnaire took approximately 30–45 min to answer.

To compensate them for their time, toiletries to the value of ZAR50 (50 South African Rands, which is equivalent to approximately 4 US$), were provided to all research participants. They were also provided with an educational session on reproductive and sexual health after they had completed the questionnaire.

After obtaining ethical clearance (IREC 97/17) from the university research ethics committee, gatekeeper permission was obtained from the director of the Dennis Hurley Centre. All participants were provided with a letter of information. Signed consent was obtained prior to answering the questionnaire. Participation was voluntary and those who did not wish to participate were not penalised in any way.

### Quantitative analysis

The statistical package SPSS (version 24) was used to analyse quantitative data. Frequencies were calculated. Data were correlated using Chi-square tests. Odds ratios were computed for binary outcome variables. Multivariate regression modelling was conducted using a backward stepwise process with the inclusion of relevant covariates. Confidence intervals (95%) were calculated and a *p*-value less than .05 was considered statistically significant.

### Qualitative analysis

Tesch’s eight steps of thematic analysis were used to analyse the qualitative data (Creswall, 2014). (1) Initially one researcher (CMK) familiarised herself with the data by reading the open-ended responses multiple times to gain insight into the content of the research. (2) The researcher manually coded the data. (3) A list of all the themes was made and similar themes clustered together. (4) All researchers reviewed the themes and revised where necessary. (5) The themes were defined and named appropriately. (6) A final decision was made by the researchers on the themes used. (7) The data were analysed and a coherent report produced. (8) The researchers recoded the data where necessary. Some quotations have been reported to affirm the data derived from the quantitative analysis. Sentences were neatened up to correct grammar, if required.

## Results

One hundred and nine questionnaires were administered. The demographics of the study participants are indicated in Table 1. All participants were over the age of 18, majority were single (76.1%; *n *= 83) and of the Black African race (85.3% *n *= 93). Half the study participants (51.4%; *n *= 56) had completed school, with no further education whilst 10.1% (*n *= 11) had not completed school education. Almost half (44.2%; *n *= 48) were unemployed and 17.4% (*n *= 19) were homeless. Of those who were homeless, 9.1% (*n *= 10) lived on the streets of the inner city and 8.3% (*n *= 9) lived in informal shelters within the city.

**Table 1.** Participant demographics.

Characteristics	*n* (%)
Age	
18–30	51 (47.8)
31–50	48 (44)
Over 50	8 (7.3)
Marital status	
Single	83 (76.1)
Married	17 (15.6)
Widowed	3 (2.8)
Divorced	2 (1.8)
Cohabiting	2 (1.8)
Ethnicity	
Black	93 (85.3)
Other	14 (12.8)
Nationality	
South African	94 (86.2)
Foreign national	12 (11)
Education level	
Incomplete schooling	11 (10.1)
Completed schooling	56 (51.4)
Tertiary education	31 (28.4)
Post-graduate training	8 (7.3)
Employment	
Employed	24 (22.0)
Street hawker/vendor	17 (15.6)
Unemployed	44 (40.4)
Pensioner	4 (3.7)
Sex worker	1 (0.9)
Type of housing	
Formal housing	71 (65.1)
Homeless	19 (17.4)

Knowledge of HIV transmission was high, with majority of participants correctly identifying that multiple concurrent sexual partners increase one’s risk of HIV acquisition (98%) and condom use decreases the risk of transmission (86.6%). In addition, participants knew that a person living with HIV can look and feel healthy (84.2%). Most correctly identified myths related to modes of transmission such as sharing utensils (88.2%) or coughing and sneezing (83.5%). Moreover, 76.5% indicated that AIDS cannot be cured.

Almost all the participants (97.1%; *n *= 99) had access to free HIV screening and 91.2% (*n *= 93) had been tested at some point. The need for regular screening was recognised with 68.8% (*n *= 64) participants tested a minimum of three times. HIV-positive status was disclosed by 13.3% (*n *= 12) while 10% (*n *= 9) did not reveal their status.

Table 2 indicates aspects of the sexual behaviour of the participants. Some (13.3%; *n *= 12) indicated that they had multiple concurrent sexual partners. Over a third of the participants (40%; *n *= 36) had their sexual debut before the age of 18 years. Only 22.2% (*n *= 20) had used a condom consistently during the last year and 14.4% (*n *= 13) indicated that condoms were not used due to fear of being assaulted by the sexual partner, should a condom be requested. Quarter of the women (26.7%; *n *= 24) had been assaulted by a sexual partner. Some (18.9%; *n *= 17) had been forced to have sexual intercourse at some stage of their lives and 10% (*n *= 9) were involved in transactional sex. Of these, all nine women had received money for sex and eight had additionally received either shelter, food, drugs, personal grooming sessions, school/university fees or a cell phone.

**Table 2.** Sexual behaviour of sexually active participants (*n* = 90).

	*n* (%)
Current sexual partners	
One	72 (80)
Multiple	12 (13.3)
Lifetime sexual partners	
1	26 (28.9)
2	18 (20)
3	15 (16.7)
More than 3	27 (30)
Age at first sexual intercourse	
13–18	36 (40)
19–21	28 (31.1)
22–28	20 (22.2)
Use of condom at every sexual act in the last year	20 (22.2)
Unable to negotiate condom use due to fear of been assaulted by sexual partner	13 (14.4)
Ever been forced to have sexual intercourse	17 (18.9)
Ever been assaulted by a sexual partner	24 (26.7)
Transactional sex	9 (10)

Chi-square tests showed no significant relationship between the HIV knowledge scores and the HIV prevention scores of participants (*p *= .457). Both bivariate and multivariate analyses showed no relationship between condom use and participation in transactional sex, having concurrent multiple sexual partners or living in informal housing indicating that those involved in risky behaviour did not use condoms despite adequate knowledge of the HIV protection offered by such use (Table 3).

**Table 3.** Bivariate and multivariate analysis exploring association between selected risk factors and condom use.

	Bivariate analysis			Multivariate analysis		
	OR	95% CI	*p-*value	aOR	95% CI	*p-*value
Transactional sex	1.00	0.19–5.24	1.00	0.72	0.09–5.58	0.75
Concurrent multiple sexual partners	1.29	0.31-5.39	0.73	1.24	0.28–5.67	0.78
Informal housing	1.34	0.38–4.78	0.65	1.59	0.34–7.47	0.55

Bivariate analysis showed an association between living in informal housing and participation in transactional sex (OR = 31.94, 95% CI: 5.65–180.63, *p *< .001) as well as multiple current sexual partners (OR = 6.30, 95% CI: 1.39–28.42, *p *= .02). Multivariate analysis, after adjusting for all other factors indicated that the odds of having transactional sex was increased by 23 times in those who did not have formal housing (OR = 23.306, 95% CI: 3.97–144.59, *p *= .001). There was no association between having multiple sexual partners and transactional sex in the multivariate model (Table 4).

**Table 4.** Bivariate and multivariate analysis for risks associated with transactional sex.

	Bivariate analysis			Multivariate analysis		
	OR	95% CI	*p-*value	aOR	95% CI	*p-*value
Condom use	1.00	0.19–5.24	1.00	0.59	0.06–5.14	0.64
Concurrent multiple sexual partners	6.30	1.39–28.42	0.02	4.05	0.63–26.23	0.14
Informal housing	31.94	5.65–180.63	<0.001	23.96	3.97–144.59	0.001

### Qualitative responses

Two themes emerged from the qualitative responses. The first theme was poverty, with the sub-themes of employment opportunities, transactional sex and lack of housing. The second theme was independence.

The women felt that poverty is the overarching factor determining their health. Alleviation of poverty would help solve other challenges. In response to the question ‘What more do you think could be done to improve the health of women in Durban?’, the responses indicated that nothing could be done since the main concern was poverty, there was less concern about health and prevention of disease.

I don’t think you can, because people do as they wish. Everybody is poverty stricken. (P38)

This discernment by participant 38 indicates that due to the extreme poverty, people did not care about anything else. Health issues were secondary to concerns such as getting a meal and shelter. They needed employment to overcome these difficulties and felt that job opportunities should be created by the government.

Government must give us jobs to become a healthy good citizen. Poverty kills us. (P95)

Increase of employment opportunities. (P5, P3, P4)

Due to the lack of employment, some women resorted to transactional sex in order to survive. The creation of employment opportunities would, to some extent, prevent these women from engaging in transactional sex.

To create job opportunities for women, so that they will stop doing prostitution. (P6)

This was re-iterated by another participant, who indicated that transactional sex engagement was due to the dire need to survive.

Providing jobs so that they won't sleep around, in order to survive. (P119)

Provision of housing was also important in alleviating poverty. It emerged that many women did not have any form of housing and sought shelter under bridges in the city.

Women should be moved from staying under the bridges and have a place built for them where they can stay. (P11)

The second theme that emerged was on independence for women. They expressed the desire not to be dependent on men. The dependence on men, which was most likely financial, led to a lack of respect towards women by men.

Durban women need to be more independent and not depend on men, because men think without them women cannot live. (P42)

Men should start respecting women and address them properly. (P57)

## Discussion

This study indicates a high level of awareness of HIV transmission (>80%) among women in the inner city of Durban, KwaZulu-Natal, South Africa. More than 90% of the participants had tested for HIV; the prevalence among the study population was 13.3%. However, the high level of knowledge did not appear to translate to behavioural change to reduce infection rates. In particular, it was noted that condom use was low, there was partner concurrency and transactional sex was also prevalent.

The high level of knowledge shown in this study is comparable to women in another vulnerable community in the city and also to that of South African women aged 15–24 years in the 2018 Global AIDS report (Haffejee et al., 2016; SANAC, 2018). In fact, there was greater awareness of multiple partners increasing the risk of transmission, from 84.2% in the latter report to 98% in the current study (SANAC, 2018). Despite this, the number of participants with multiple concurrent sexual partners was high, with almost a fifth of those who considered themselves to be in a steady relationship, having multiple concurrent sexual partners. Other studies in South Africa also attest to a high prevalence of multiple concurrent sexual partners (Haffejee & Maksudi, 2020; Haffejee, Fasanmi-Kana, Ally, Thandar, & Basdav, 2022; Haffejee, Koorbanally, & Corona, 2018b), which is associated with a greater prevalence of HIV as it creates additional interconnected sexual networks (Kenyon, Tsoumanis, Schwartz, & Maughan-Brown, 2016).

Moreover, one-tenth of the sexually active participants engaged in transactional sex, sometimes for basic goods, such as food and shelter. Financial and social difficulties force women into sexual relationships to meet their basic needs. As the women attested in the qualitative responses, poverty was the overarching factor in the lives of these women. With an unemployment rate of 40%, these women find other means to survive. Basic survival needs, particularly among those who have limited economic opportunities, compels individuals to enter into sexual relationships, which are of a transactional nature, where sex is used as a resource to trade for basic household and personal items (Bernays et al., 2021). As the DHC provides services to the homeless population of the area, with one-tenth of the study participants reporting themselves as homeless and living on the street, a sexual relationship of a transactional nature provides shelter and food for at least some of the time as affirmed by both the quantitative data and the qualitative interviews. These relationships tend to be transient in nature and usually concurrent, increasing the risk for HIV infection (Bernays et al., 2021). While there is no accurate census data on homeless rates in South Africa, the Human Sciences Research Council estimates approximately 200,000 homeless street people in South Africa (Rule-Groenewald, Timol, Khalema, & Desmond, 2015), a socio-economic factor that serves as a barrier in implementing the desired behavioural change.

These relationships often have an extreme power discrepancy (Hunter, 2002) where women feel compelled to enter into these relationships due to economic challenges their negotiating power for safe sex is negligible (Fehr, Vidourek, & King, 2015; Haffejee & Maksudi, 2020). The risk of HIV acquisition is ‘traded-off’ in favour of economic gain (Bernays et al., 2021). Negotiating power is also diminished in abusive relationships, which were confirmed by almost a quarter of the women in the current study. This could explain that despite an 80% awareness that condoms prevented HIV transmission, only 22.2% of the sexually active participants consistently used these. In addition, 15% of participants were afraid of assault, should they insist on the use of a condom. When weighing up the pros and cons of condom use, for many women, the condom was just not worth it.

In circumstances where money or goods are exchanged, the reward is often higher for riskier sexual practice (Saasa et al., 2018). Other studies have also linked gender-based violence (GBV) with difficulty in negotiating safe sex and therefore inconsistent condom use with the resultant unwanted pregnancies and HIV infection (Dunkle et al., 2004; Gass, Stein, Williams, & Seedat, 2010; Lang, Salazar, Wingood, DiClemente, & Mikhail, 2007). These factors are further compounded by relationship power inequity or gender inequality which is prevalent in South Africa (Zembe, Townsend, Thorson, Silberschmidt, & Ekstrom, 2015). The link between HIV and intimate partner violence is to be expected as they share common social and economic risk factors including poverty, limited economic opportunities, high-risk sexual norms, as well as gender inequalities (Zembe et al., 2015).

The increased risk of HIV infection associated with more violent sexual encounters is of concern in the current population, with almost a fifth of the women having been forced to have sex. While violence against women, is a worldwide phenomenon and human rights concern, South Africa has one of the highest rates of violence against women globally (CADRE, 2003). This is most commonly perpetrated by an intimate partner (IPV) with studies in eThekwini reporting almost 50% of women experiencing IPV (Interim Steering Committee, 2020). Gender-based violence and specifically IPV, have a direct negative impact on safe sexual practices (Gaffoor, Wand, Daniels, & Ramjee, 2013).

Overall, the high level of risky sexual behaviour can be explained in terms of the COM-B model of behaviour, which explains that capacity, opportunity and motivation interact to produce a behaviour. The capacity to enact a particular behaviour includes aspects like knowledge of the required behaviour. Opportunity to endorse the behaviour can be either social or physical and is generally outside of the individual control, while motivation may be automatic, in the form of habit or reflective in the form of choice (Michie, Stralen, & West, 2011). In this study population, the level of knowledge of HIV was high as reflected by the correct responses to HIV transmission and prevention. However, the opportunity and motivation for decreasing risky sexual behaviour were low, largely due to poverty and its associated effects. The struggle to survive in the face of a current lack of basic supplies like food and shelter, outstrips the fear of possible HIV infection that they would deal with at a later stage. Indeed, in a South African case study, Stoebenau et al. (2011) found that the fear of contracting HIV, did not translate into safer sexual practice. In fact, they found that HIV infection had become normalised as something that one has to come to terms with. Macintyre, Brown, and Sosler (2001) found that behaviour change in African communities was partly determined by the level of mortality experienced, and that the extension of life expectancy with antiretroviral drugs (ARVs) may also have resulted in increased risky sexual behaviour (Saasa et al., 2018).

In the open-ended responses participants indicated that without employment they would not be empowered to make better decisions regarding their health. Furthermore, they requested job opportunities, so that women could be financially independent, with a safe place to live in.

Notwithstanding the high level of knowledge of HIV transmission, 34.9% of participants did not feel that they had enough information about HIV and wanted more education drives. The high level of screening (90%) also shows an understanding of the importance of knowing one’s status as well as the easy access to free testing. Similar findings emanated from a study in Uganda, however, they report that testing is often poorly timed as it occurs within a few days of engaging in risky sexual behaviour and may therefore occur within the virus window period (Bernays et al., 2021). Nevertheless, screening was current, with 64.2% tested within the previous year which was significantly higher than reported four years previously, where only 50% of residents, within a vulnerable community, had tested in the preceding year (Haffejee et al., 2016). Testing centres should provide opportunities for discussions around safe sex, particularly for those who test negative, so that they remain negative (Bernays et al., 2021). HIV prevention packages should incorporate the cultural, biological, socio-economic and structural aspects women face. The potential use of biomedical prevention such as pre-exposure prophylaxis (PrEP) may assist in a power-shift as this can be termed as ‘women-controlled prevention’ (Collier, Colarossi, & Sanders, 2017). This option will be of particular importance to women who are not in the position to negotiate condom use, particularly when there is intimate partner violence or transactional sex.

Education drives and screening by themselves are insufficient to bring about change. As gauged from the qualitative interviews, there was a struggle to survive with the ensuing dependence on men, which led to some women enduring abusive relationships and others engaging in transactional sex, both of which put women at risk of HIV acquisition. Behavioural changes to decrease infection rates are required but these may only be possible with an alleviation of poverty and empowerment of women. When suggesting HIV prevention methods, understanding the impeding factors women face is important for its success, until such a time when policies and funding opportunities are in place to alleviate poverty and provide much-needed financial independence among women.

## Limitations

Due to the length of the questionnaire and the number of participants, research assistants were not able to fully explore the lived experiences of the participants in the qualitative part of the study. In addition, the data were self-reported which could lead to bias towards socially acceptable responses. Finally, this study focused only on women from a marginalised community as this was part of a larger study held at a wellness day to provide health services to women from this community and are not generalisable. Future studies should include data on the behavioural practices of heterosexual men as this could provide useful information on the spread of HIV in the region.

## Conclusion

HIV health behaviour is not determined by knowledge alone. In South Africa, the main benefit from the education campaigns is the increased awareness of HIV screening. Although, participants from this study understood the required behaviour change to prevent HIV transmission, this vulnerable group does not have the opportunity nor the motivation to enact such behaviour change. In the current climate of increasing unemployment and escalating GBV, urgent interventions are needed in terms of employment opportunities and empowerment drives to prevent an increase in HIV transmission.

## Data Availability

Data will be available on reasonable request from the corresponding author.
